# Teriflunomide Is an Indirect Human Constitutive Androstane Receptor (CAR) Activator Interacting With Epidermal Growth Factor (EGF) Signaling

**DOI:** 10.3389/fphar.2018.00993

**Published:** 2018-10-11

**Authors:** Alejandro Carazo, Jan Dusek, Ondrej Holas, Josef Skoda, Lucie Hyrsova, Tomas Smutny, Tomas Soukup, Martin Dosedel, Petr Pávek

**Affiliations:** ^1^Department of Pharmacology and Toxicology, Faculty of Pharmacy, Charles University, Prague, Czechia; ^2^Faculty of Medicine and Dentistry, Institute of Molecular and Translational Medicine, Palacky University, Olomouc, Czechia; ^3^Department of Pharmaceutical Technology, Faculty of Pharmacy, Charles University, Prague, Czechia; ^4^Division of Rheumatology, 2nd Department of Internal Medicine – Gastroenterology, Faculty of Medicine, University Hospital in Hradec Kralove, Charles University, Prague, Czechia; ^5^Department of Social and Clinical Pharmacy, Faculty of Pharmacy, Charles University, Prague, Czechia

**Keywords:** CAR, nuclear receptor, gene regulation, cytochrome P450, metabolism

## Abstract

The constitutive androstane receptor (CAR) is a nuclear receptor involved mainly in xenobiotic and endobiotic metabolism regulation. CAR is activated directly by its ligands via the ligand binding domain (LBD) or indirectly by inhibition of the epidermal growth factor (EGF) signaling. We found that leflunomide (LEF) and its main metabolite teriflunomide (TER), both used for autoimmune diseases treatment, induce the prototype CAR target gene *CYP2B6* in primary human hepatocytes. As TER was discovered to be an EGF receptor antagonist, we sought to determine if TER is an indirect activator of CAR. In primary human hepatocytes and in differentiated HepaRG cells, we found that LEF and TER up-regulate CAR target genes *CYP2B6 and CYP3A4* mRNAs and enzymatic activities. TER stimulated CAR+A mutant translocation into the nucleus but neither LEF nor TER activated the CAR LBD, CAR3 variant or pregnane X receptor (PXR) in gene reporter assays. Interestingly, TER significantly up-regulated CAR mRNA expression, a result which could be a consequence of both EGF receptor and ELK-1 transcription factor inhibition by TER or by TER-mediated activation of glucocorticoid receptor (GR), an upstream hormonal regulator of CAR. We can conclude that TER is a novel indirect CAR activator which through EGF inhibition and GR activation controls both detoxification and some intermediary metabolism genes.

## Introduction

The nuclear receptor constitutive androstane receptor (CAR; also known as nuclear receptor subfamily 1, group I, member 3, NR1I3), a member of the nuclear steroid and thyroid hormone receptor superfamily, is a transcription factor that is involved in the regulation of key xenobiotic and intermediary metabolism genes. CAR has been recognized as a xenobiotic-sensing nuclear receptor that transcriptionally regulates the expression of detoxification enzymes and transporters involved in the metabolism and elimination of endogenous and exogenous substances such as bilirubin, steroids, and xenobiotics (di Masi et al., 2009). Recently, proof of the important role of CAR in the regulation of lipid, glucose and bile acids metabolism has emerged (Yan et al., 2015). The human CAR displays unique properties in comparison with other nuclear receptors or with its rodent orthologs, including high constitutive activity, both direct LBD-dependent and LBD-independent activation, and spontaneous nuclear localization in tumor cell lines (Molnar et al., 2013; Mackowiak and Wang, 2016).

Constitutive androstane receptor is inactive in the cytoplasm of hepatocytes as homodimer phosphorylated at Thr^38^ within its DNA binding domain (DBD). In this state, CAR physically interacts with ERK1/2 kinase near the C terminus of CAR. This binding enables CAR to form its homodimer and prevents CAR from binding to PP2Ac, thereby retaining CAR phosphorylation and cytoplasmic localization. CAR activation by a CAR ligand or ERK1/2 inhibition stimulate CAR homodimer dissociation to monomers. PP2Ac utilizes the receptor for activated C kinase 1 (RACK1) as a regulatory subunit to dephosphorylate CAR monomer at Thr^38^. Then, the nonphosphorylated CAR monomer translocates into the nucleus to interact with the regulatory promoter regions as a heterodimer with RXRα (Mutoh et al., 2009, 2013).

Both ligand binding cavity-dependent and indirect activation of human CAR has been described as releasing CAR from its cytoplasmic tethering complex (Chai et al., 2016; Mackowiak and Wang, 2016; Shizu et al., 2017). The agonistic ligand 6-(4-chlorophenyl) imidazo[2,1-*b*]thiazole-5-carbaldehyde *O*-(3,4-dichlorobenzyl) oxime (CITCO) binds directly to the cytoplasmic CAR homodimer and dissociates the phosphorylated human CAR (hCAR) into its monomers, exposing the PP2A/RACK1 binding site for subsequent dephosphorylation of CAR at Thr^38^. Phenobarbital, which is not a direct CAR ligand, but reverses the epidermal growth factor (EGF) signal at the EGF receptor, triggers the dissociation of down-stream kinase ERK1/2 from CAR homodimer and converts it into monomers eligible for desphosphorylation at Thr^38^ by PP2Ac (Mutoh et al., 2013; Shizu et al., 2017).

Important insights into this indirect activation mechanism of CAR have been obtained recently based on the finding that PB, the classic CAR-activating drug, antagonizes EGF signaling by direct binding to the epidermal growth factor receptor (EGFR). In agreement with this theory, erlotinib, an EGFR antagonist, has been found to dissociate CAR homodimer, dephosphorylates CAR and significantly induces *Cyp2b10* mRNA in primary mouse hepatocytes (Shizu et al., 2017). Recently, we described that flavonoids galangin, chrysin, and baicalein stimulate EGFP-tagged hCAR nuclear translocation and *CYP2B6* mRNA induction in primary human hepatocytes, although they do not interact with the CAR LBD. Consistently, all these flavonoids repress EGFR autophosphorylation at Tyr^1068^ and phosphorylation of downstream transcription factor ELK1 after EGF treatment (Carazo Fernandez et al., 2015).

**FIGURE 1:**
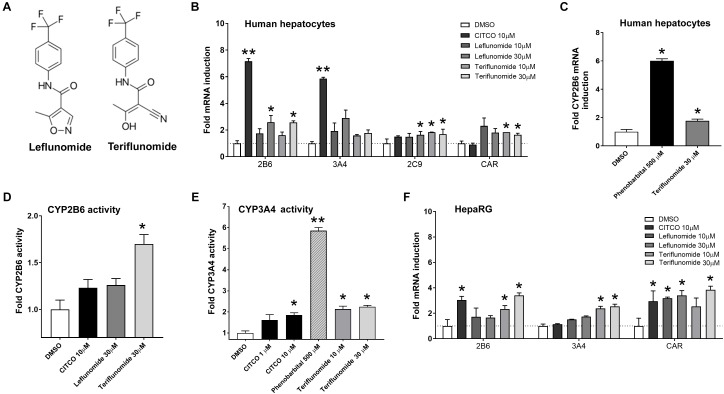
Leflunomide and teriflunomide enhance the mRNA expression of hCAR target genes *CYP2B6*, *CYP3A4*, and *CYP2C9*, and CAR mRNA in human hepatocytes and HepaRG cells. **(A)** Structure of LEF and TER. **(B)** Primary human hepatocytes from four donors **(B–F)** differentiated HepaRG cells were treated with CITCO, phenobarbital, LEF and TER for 48 h before isolation of mRNA and qRT-PCR analyses. Data are presented as the mean ± SD of fold mRNA upregulation from three independent donors or experiments (*n* = 3) and compared to a vehicle sample (0.1% DMSO) set to 1 **(B,F)**. Induction experiments in panels C have been perform in primary human hepatocytes (batch HEP220990) **(D,E)** CYP2B6 and CYP3A4 catalytic activities after treatment with CITCO (10 μM), phenobarbital (500 μM), TER and LEF (30 μM) for 48 h was examined using the P450-Glo^TM^ CYP2B6 and CYP3A4 Assays with luciferin 2B6 and luciferin IPA in primary human hepatocytes (batches HEP220913 and HEP220990) ^∗^*P* < 0.05, ^∗∗^*P* < 0.05 – statistically significant difference compared to the control (DMSO-treated) cells (ANOVA with a Dunnett’s *post hoc* test).

Leflunomide (LEF) belongs to the Disease Modifying Antirheumatic Drugs (DMARDs), and it is used for the treatment of rheumatoid arthritis to slow down disease progression. Upon oral administration, the drug is quickly metabolized to teriflunomide (TER) by the opening of the isoxazole ring (**Figure 1A**). TER has been found as inhibitor of EGF receptor and several synthetic analogs have been developed as potential EGFR tyrosine kinase inhibitors for breast cancer therapy (Ghosh et al., 1998, 1999).

In the present work, we aimed to study the mechanism by which LEF and TER induce *CYP2B6* mRNA as well as whether these compounds are activators or ligands of CAR receptor. We found that TER is an indirect CAR activator via EGFR inhibition, which up-regulates hCAR expression, facilitates the nuclear translocation of the CAR+A mutant and promotes the CAR-mediated transactivation of both xenobiotic and endogenous metabolism genes. In addition, we show that TER might have a secondary mechanism through GR activation and GR-mediated hCAR induction.

## Materials and Methods

### Materials

Leflunomide, teriflunomide, 6-(4-chlorophenyl) imidazo[2,1-b][1,3]thiazole-5-carbaldehyde O-(3,4-dichlorobenzyl)oxime (CITCO), rifampicin, dexamethasone, 3-methylcholanthrene, DMSO, erlotinib, U0126, RU486, dexamethasone, phenobarbital, Hoechst 33324 dye and FBS were purchased from Sigma-Aldrich (St. Louis, MO, United States). Cell culture media (DMEM) were purchased from Thermo Fisher Scientific (Waltham, MA, United States). The final concentration of DMSO in the culture media was 0.1% (v/v) in all experiments.

### Cell Culture and CYP2B6 and CYP3A4 Enzymatic Assays

Human hepatocellular carcinoma HepG2, COS-1 (SV40 transformed African green monkey kidney), and A431 squamous carcinoma cell lines were purchased from the European Collection of Cell Cultures (ECACC, Salisbury, United Kingdom), and were maintained in antibiotic-free DMEM medium complemented according to the protocols in the ECACC data sheets. Human long-term hepatocytes in monolayer (Batch HEP220900, HEP220913, HEP220926, and HEP220990, Biopredic International, Saint-Grégoire, France) were purchased from Biopredic International (Saint-Gregoire, France), and were maintained in Biopredic basal medium with additives. Isolations were prepared from the livers of a 68 years old Caucasian male, a 69 years old Caucasian male, a 47 years old Caucasian female and 69 years old Caucasian male.

HepaRG^TM^ cells were purchased from Life Technologies (Carlsbad, CA, United States, now Thermo Fisher Scientific, Waltham, MA, United States) and were cultivated to differentiate prior to treatment as we described in our previous paper (Hyrsova et al., 2016). In order to upregulate PGC1α in primary human hepatocytes or HepaRG cells, forskolin (FSK, 10 μM) pre-treatment was performed for 1 h prior to 24 h treatment with the tested compounds. FSK treatment increased the expression of G6Pase and PEPCK1 mainly via the cAMP-response element-binding (CREB) protein-mediated induction of PGC1α, a cofactor induced under diabetic or fasting conditions (Gao et al., 2015).

The P450-Glo^TM^ CYP2B6 Assay with luciferin 2B6 or CYP3A4 P450-Glo Assays with Luciferin-IPA for cellular experiments was obtained from Promega (Hercules, CA, United States) and used according the manufacturer’s protocol in primary human hepatocytes (Batch HEP220913 and HEP220990, respectively).

### Translocation Assay

COS-1 cells were seeded on a 48-well plates (80,000 cells/well) and were incubated for 24 h. The cells were transfected (Lipofectamine^®^ 3000) with 100 ng/well pEGFP-hCAR+Ala construct. The cells were treated 24 h after transfection with CITCO (10 μM), erlotinib (10 μM), phenobarbital (500 μM), LEF, and TER (30 μM), or vehicle (0.1% DMSO). For final counting cells were stained with Hoechst 33342 (10 μg/ml, for 5 min in CO_2_ incubator). Confocal microscopy was performed at 0, 24, and 48 h after treatment with a Nicon Ti microscope and Nikon A1 plus camera (Nikon, Japan) using 405 and 488 nm lasers. The pinhole diameter was set at 35.76 μM and microphotographs were taken using NIS Elements AR 4.20 software (Laboratory Imaging, Czechia). Four photographs of every treatment were taken and cytoplasmic, nuclear and mixed localization was determined in at least 100 cells in three independent experiments (*n* = 3). pEGFP-hCAR construct kindly donated by Dr. Y. Kanno (Kanno et al., 2007) was used for site directed mutagenesis (GeneArt^TM^ Site-Directed Mutagenesis System, Thermo Fisher Scientific, Waltham, MA, United States) to insert extra alanine at position 271 of the hCAR LBD with primers 5′-CCCTCTTCTCTCCTGCTGACCGACCTGGAGTTAC-3′ and 5′-GTAACTCCAGGTCGGTCAGCAGGAGAGAAGAGGG-3′ as previously described (Chen et al., 2010).

### CAR Assembly Assay

Constitutive androstane receptor LBD assembly assays were performed with constructs and using the protocol described in our previous report (Carazo Fernandez et al., 2015). Briefly, hCAR LBD was transfected into HepG2 cells using two constructs encoding C (151–349 aa, helices 3–12) and N (103–150 aa, helix 1) terminal parts together with the pGL5-luc luciferase reporter plasmid (Promega) containing UAS binding domain and pRL-TK *Renilla* plasmid. In this set-up, a ligand promotes interaction of the helix 1 and helices 3-12, resulting in coactivator recruitment and luciferase synthesis. Lipofectamine^®^ 3000 was used as a transfection reagent according to the manufacturer’s protocol.

### Gene Reporter Assays

All transient transfection reporter gene assays were performed with Lipofectamine^®^ 3000 (Life Technologies, Carlsbad, CA, United States, now Thermo Fisher Scientific, Waltham, MA, United States) in HepG2 or A431 cells as we described in our previous reports (Rulcova et al., 2010; Krausova et al., 2011). The HepG2 cells were seeded at a density of 40,000 cells/cm^2^ onto 48-well plates 24 h before transfection. The cells were transfected with 150 ng/well luciferase reporter gene constructs (p3A4-luc, pGRE-luc, p1A1-luc, pGL5-luc, or p2B6-luc) together with 100 or 200 ng/well of a corresponding nuclear receptor expression vector (pSG5-hSXR, pSG5-hGRα, or CAR3) and pRL-TK *Renilla* construct for transfection normalization (30 ng/well). The cells were treated for 24 h with compounds in indicated concentrations before cell lysis, and firefly and Renilla luciferase analyses (Dual Reporter assay, Promega, Hercules, CA, United States). The expression plasmid pSG5-hPXR for the human PXR receptor was kindly provided by Dr. S. Kliewer (University of Texas, Dallas, TX, United States). The p2B6-luc (originally termed as B-1.6k/PB/XREM) was kindly donated by Dr. Hongbing Wang (University of Maryland School of Pharmacy, Baltimore, MD, United States). Expression construct for ligand-activated hCAR receptor transcription variant 3 (CAR3) was a kind gift from Dr. C. J. Omiecinski (Pennsylvania State University, State College, PA, United States). A phospho-defective GR (GR K419) mutant resistant to ligand-induced proteasome-mediated degradation was obtained from Dr. Zdenek Dvorak (Palacky University, Czechia). In all nuclear receptors, m and h denote mouse and human, respectively.

### TR-FRET CAR and GR Coactivator Binding Assay

The LanthaScreen^®^ TR-FRET CAR Coactivator Binding Assay (Thermo Fisher Scientific, Waltham, MA, United States) was performed according to the manufacturer’s protocol with some modifications described in our previous paper (Carazo Fernandez et al., 2015). The LanthaScreen^®^ TR-FRET GR assay was performed following the protocol of the manufacturer. Results were obtained after fluorescence signal analysis with appropriate filters using the Synergy Biotek plate reader (BioTek Instruments Inc., Winooski, VT, United States). Results were calculated as relative fold interaction of a nuclear receptor with the coactivator fragment to the control (vehicle-treated, DMSO 0.1%) signal set to 1. Data are presented as the means and SD of three different experiments (*n* = 3) performed in quadruplicates.

### qRT-PCR

The HepaRG cells were seeded in 12-well plates (26,000 cells/cm^2^) and cultivated until differentiation for 4 weeks. RNA was isolated from the HepaRG cells and human hepatocytes using TRIZOL^®^ (Invitrogen/LifeTechnologies, Carlsbad, CA, United States). cDNA was synthesized using Tetro cDNA Synthesis kit (Bioline, London, United Kingdom). The qRT-PCR procedure is detailed in our previous paper (Krausova et al., 2011). The cells were treated without transfection with LEF or TER (10 and 30 μM), CITCO (5 or 10 μM) and the vehicle control (DMSO 0.1%) for 48 h. qRT-PCR reaction mixtures contained BHQ1-FAM probes to detect expression of the *HPRT* (a housekeeping gene), *CYP2B6, CYP3A4, CYP2C9*, and *CAR* genes (Generi Biotech, Hradec Králové, Czechia). G6Pase (G6PC), PEPCK1, FASN, and HMGCS2 TaqMan probes were obtained from Life Technologies (Thermo Fisher Scientific, Waltham, MA, United States). Data are presented as the fold mRNA induction in gene expression related to the vehicle control (DMSO) set to 1, from at least three independent experiments (*n* = 3) performed in triplicates.

### Elk1 Activation Assay

MAPK/ERK cascade activation was measured using the PathDetect Elk1 trans-Reporting System (Agilent Technologies, Inc., Santa Clara, CA, United States), with a pFA2-ELK1 construct as we have described before (Carazo Fernandez et al., 2015). Elk1 transcription factor was used as a relatively specific downstream factor of EGFR activation. A431, a model cell line with high expression of EGFR receptors, was used. The cells were seeded at the density concentration of 30,000 cells/cm^2^ and transfected after 24 h with pGL5-luc luciferase reporter construct (150 ng/well), expression plasmid pFA2-ELK1 (100 ng/well) and *Renilla reniformis* luciferase plasmid (30 ng/well). After overnight incubation, the cells were washed with PBS and treated with EGF (50 ng/ml) in combination with the tested compounds, reference compounds (U0126, phenobarbital, erlotinib or dexamethasone) or vehicle (0.1% DMSO), respectively. The luminescence signal was measured with a Dual Luciferase Reporter Assay (Promega, Madison, WI, United States). Data are expressed as relative activation of firefly luciferase (normalized with *Renilla* luciferase) in each sample relative to the EGF-treated control, set at 100%.

### EGFR Phosphorylation Assays

EGFR phosphorylation at Y1068 after activation with EGF was measured in A431 cell lysates by the sandwich ELISA method following the manufacturer’s protocol (Human Phospho-EGFR (Y1068) DuoSet IC ELISA, Bio-Techne, Minneapolis, MN, United States). A431 cells were seeded at the density concentration of 200,000 cells/cm^2^. The cells were pre-treated for 30 min with serum-free OptiMEM^TM^ medium and then treated for 30 min with LEF and TER (1, 5, or 10 μM), CITCO and erlotinib (10 μM), or with phenobarbital (500 μM). After the period, an additional 10 min treatment with EGF (50 ng/ml) followed, after which the cells were analyzed using anti-phospho-EGFR (Y1068) and unphosphorylated EGFR antibodies according to the manufacturer’s instructions. Absorbance was measured at 450 nm and 540 nm, respectively, and the background signal was subtracted. A control EGF-treated cell sample was assigned as 100% activity.

### Molecular Modeling

Docking simulations were carried out using MOE (version 2016.0802) software (CCG, Montreal, QC, Canada). The crystal structure of hCAR was prepared using PDB structure 1XV9 chain B as starting geometry. This geometry was selected because the molecular characteristics of both LEF and TER are more similar to that of the pregnane-3,20-dione rather than CITCO. Pregnane-3,20-dione and the nonconserved water molecules were removed from the model and energy minimization was performed in order to relieve energy strain within the protein molecule (Gradient: 0.001 RMS kcal/mol/A^∧^2). An MOE site finder was used for the localization and mapping of an active site. Ligand structures (leflunomide and Z-teriflunomide) were prepared using the Builder Tool MOE (version 1.0). Hydrogens were added to the prepared ligand structures, partial charges were assigned (AMBER94 Forcefield), and energy minimized. Ligands were subsequently docked using the placement described in our previous paper (Dusek et al., 2017). Docking set-up was verified by the re-docking of pregane-3,20-dione into its original position. The RMSD of the re-docked pose was 0.02 Å. Ligand molecules were placed (Triangle Matcher), and the energy of the placed pose was calculated (Affinity dG), refined and rescored (GBVI/WSA dG).

### Statistical Analysis

All data are presented as the mean ± standard deviations (SD). A one-way analysis of variance (ANOVA) with a Dunnett’s *post hoc* test was applied. All statistical analyses were performed using GraphPad Prism 6 Software (GraphPad Software, Inc., San Diego, CA, United States) based on at least three independent experiments (*n* = 3). A *p*-value of <0.05 was considered to be statistically significant.

## Results

### Leflunomide and Its Metabolite Teriflunomide Up-Regulates CAR Target Genes in Human Hepatocytes and HepaRG Cells

To determine the effect of LEF and TER (**Figure 1A**) on the gene expression of key cytochrome P450 enzymes regulated by hCAR receptor, human hepatocytes and differentiated HepaRG cells were treated with vehicle (control, 0.1% DMSO), CITCO (10 μM), phenobarbital (500 μM), or LEF and TER in concentrations of 10 and 30 μM for 48 h. Significant mRNA induction was observed for all genes (*CYP2B6, CYP3A4, CYP2C9* as well as for hCAR) in a dose-dependent manner in the human hepatocytes (**Figures 1B,C**). In agreement with these findings, we found a significant increase in CYP2B6 and CYP3A4 catalytic activity after treatment with TER for 48 h in primary human hepatocytes (**Figures 1D,E**). The effect of TER was even more pronounced than the effect of CITCO. A similar expression profile of CAR target genes was obtained in HepaRG cells (**Figure 1F**).

### Teriflunomide Stimulate Cytoplasm-Nuclear Translocation of EGFP-Tagged CAR+Ala

**FIGURE 2:**
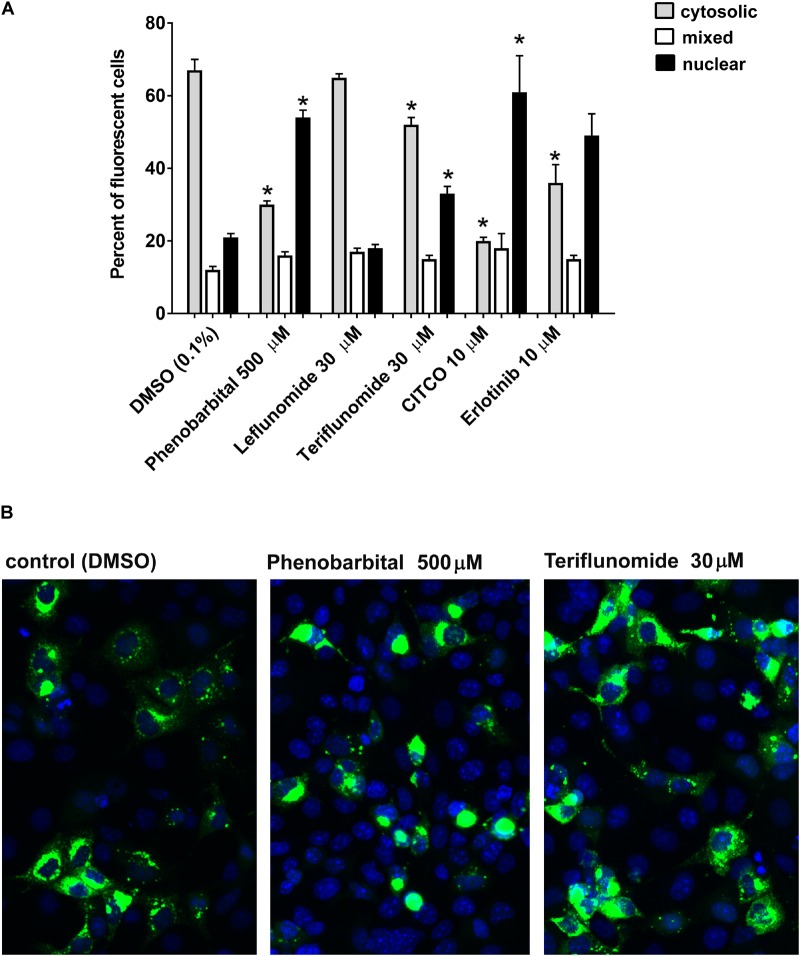
Drug-stimulated cytoplasm-nuclear translocation of hCAR ligand-sensitive mutant (hCAR+Ala) in COS-1 cells. **(A)** COS-1 were transfected with 100 ng/well of pEFGP-CAR+Ala mutant and exposed to phenobarbital, LEF, TER, CITCO, or EGFR antagonist erlotinib for 24 h. After incubation, the cells were stained by Hoechst 33342. Confocal microscopy was performed, and cells with dominant cytoplasm, nuclear or mixed (nuclear and cytoplasm) fluorescence localization were classified. At least 100 cells were analyzed for each compound and percentage calculated in three independent experiments (*n* = 3). **(B)** TER partly simulates nuclear localization of EGFP-tagged hCAR+Ala, although the majority of fluorescence remains in the cytoplasm after 48 h. Confocal images illustrate representative localization and translocation of EGFP-tagged hCAR+Ala expression after the vehicle control or drug treatment. Magnification 20×. ^∗^*P* < 0.05 – statistically significant difference compared to nuclear or cytoplasm localization in the control (DMSO-treated) cells (ANOVA with a Dunnett’s *post hoc* test).

Next, we examined if LEF and TER promotes the nuclear translocation of the CAR+Ala mutant in COS-1 cells as was shown before (Chen et al., 2010). This CAR mutant displays reduced basal activity and can be significantly activated by direct ligand activation to translocate into the nucleus. Cellular fluorescence expression was classified into cytoplasmic, nuclear, or mixed (cytoplasmic and nuclear) localization, with data expressed as the percentage of all fluorescent cells. We observed that CITCO as well as EGFR antagonists phenobarbital and erlotinib significantly promote cytoplasm-nuclear translocation of EGFP-tagged CAR+Ala (**Figure 2A**). We also observed that TER (30 μM) promoted a shift in cytoplasm fluorescence from 68% in the control to 52% after 24 h (**Figures 2A,B**). Nuclear localization increased from 21% in control to 33% in TER treated samples. LEF had no significant effect on EGFP-CAR+Ala translocation. Interestingly, we observed that nuclear translation happens within the first 24 h; further exposure to the tested compound did not simulate nuclear translocation of CAR in COS-1 cells.

### Leflunomide and Teriflunomide Do Not Directly Activate CAR and PXR

**FIGURE 3:**
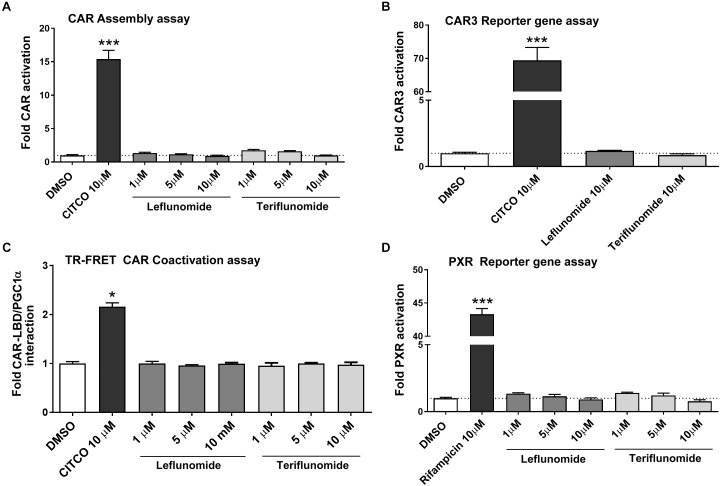
Neither leflunomide nor teriflunomide directly activate hCAR and PXR. The CAR assembly assay **(A)**, CAR3 variant reporter gene assay **(B)**, TR-FRET CAR Coactivator assay **(C)** and PXR-sensitive luciferase reporter gene assay **(D)** were performed. Luciferase reporter gene assays were performed in HepG2 cells transiently transfected with either human CAR-N, CAR-C, CAR3 variant or human PXR expression plasmids and appropriate responsive luciferase reporter promoter constructs (pGL5-luc, p2B6-luc, p3A4-luc) together with *Renilla* expression construct for transfection normalization (see section Materials and Methods). HepG2 cells were treated with LEF or TER (1, 5, or 10 μM) together with CITCO (10 μM) and rifampicin (10 μM), respectively, for 24 h. Relative activation was calculated relative to vehicle-treated samples (DMSO 0.1%) and data are presented as the means ± SD from three independent experiments (*n* = 3) performed in triplicates. **(C)** The LanthaScreen^®^TR-FRET CAR coactivator binding assay was employed to determine interactions of tested compounds with the hCAR-LBD. The manufacturer’s protocol was followed with several modifications described in section Materials and Methods. Data are presented as the relative fold interaction between hCAR-LBD and PGC1α fragment to the control (DMSO-treated) samples, set to 1. Data indicate the mean ± SD of three independent assays (*n* = 3) performed in quadruplicates. ^∗^*P* < 0.05, ^∗∗∗^*P* < 0.001 – statistically significant difference compared to the control (DMSO-treated) cells.

In the next experiments, we sought to determine if LEF and TER interact with the hCAR LBD. For this purpose, we performed a CAR assembly assay as well as an assay with a ligand-activated CAR3 variant. As shown in **Figures 3A,B**, LEF and TER were unable to significantly activate hCAR in the human HepG2 cell line after testing with all the transient transfection reporter gene assays used. In addition, neither TER nor LEF activate the pGAL4-CAR+aaa ligand-responsive CAR construct (*data not shown*). Subsequently, we studied the behavior of these compounds in a TR-FRET CAR coactivator assay with recombinant hCAR LBD protein. This assay is highly sensitive and allows discrimination between direct and indirect CAR activation, since cellular signaling is avoided under *in silico* conditions. No direct activation of the hCAR-LBD was observed by LEF or TER, confirming our previous results obtained in the cell-based assays (**Figure 3C**). In addition, we confirmed that neither phenobarbital not erlotinib significantly activate the hCAR-LBD in the TR-FRET CAR coactivator assay (Carazo and Pavek, 2015), nor in the CAR assembly or CAR3 activation assays (*data not shown*).

Bearing in mind these results, we aimed to study the effect of LEF and TER on PXR, which also transactivates *CYP2B6*, *CYP3A4*, and *CYP2C9* genes in primary human hepatocytes. However, no interaction of either LEF or TER with PXR was observed in these experiments, which used the CYP3A4-luc luciferase reporter construct in HepG2 cells cotransfected with human PXR expression vector (**Figure 3D**).

We also found that erlotinib did not activate the human CAR receptor in the CAR assembly assay, but slightly activated the CAR3 variant in the other gene reporter assay (**Supplementary Figure S1**). In addition, erlotinib significantly (*p* < 0.01) activated PXR at 20 μM concentration, and induced CYP2B6 mRNA expression in the primary human hepatocytes (**Supplementary Figure S1**).

### Leflunomide and Teriflunomide Do Not Match the hCAR LBD Cavity

**FIGURE 4:**
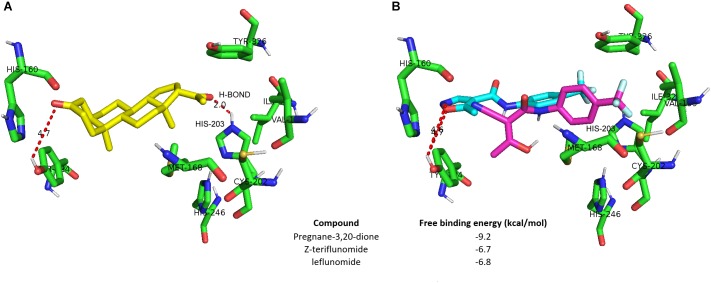
Neither leflunomide nor teriflunomide dock to hCAR LBD. Molecular docking into the crystal structure of hCAR was performed using PDB structure 1XV9 chain B. **(A)** Structure of the docked binding pose of pregnane-3,20-dione (green). **(B)** Leflunomide (blue) and *Z*-enol form of terflunomide (magenta) do not form hydrogen bonding (H-BOND) with His203, which is important for the pose stabilization of pregnane-3,20-dione in hCAR cavity.

In the following *in silico* docking experiments, we aimed to verify if LEF and TER interact with the hCAR LBD cavity. The top scored poses for pregnane-3,20-dione, LEF and the *Z*-enol form of TER are depicted in **Figures 4A,B**. Based on the free binding energy results, it is clear that both LEF and TER show a weak affinity toward the hCAR binding cavity (−6.8 kcal/mol and −6.7 kcal/mol compared to pregnane-3,20, dione, −9.2 kcal/mol, respectively). A plausible explanation for this phenomenon is the presence of triflouromethyl moiety in both the LEF and TER structure. This highly hydrophobic moiety protrudes toward the hydrophilic region formed by the residues of Met168, Val199, Cys 202, His203, His246, and Tyr326. Interactions with these residues further stabilize the pose of pregnane-3,20-dione; however, this stabilization proved impossible for both LEF and TER. Hydrogen bonding with His203 seems to be the most important factor for pose stabilization of pregnane-3,20-dione, but both LEF and TER are incapable of such stabilization owing to the steric hindrance of the trifluoromethyl moiety (**Figure 4B**).

### Leflunomide and Teriflunomide Significantly Inhibit the ELK1 Transcriptional Activation and Autophosphorylation of EGFR

**FIGURE 5:**
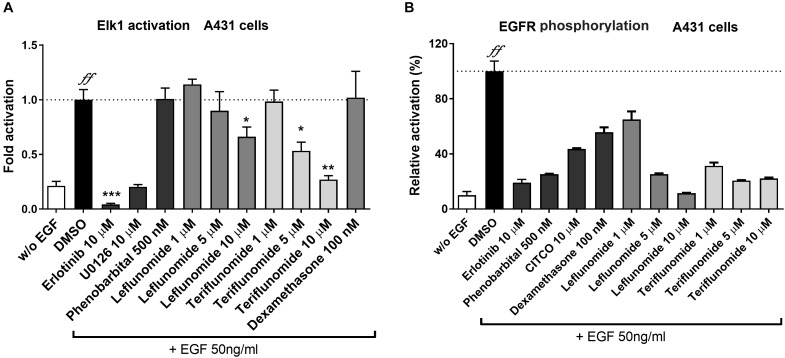
Effects of leflunomide and teriflunomide on ELK1 activation and EGFR phosphorylation in A431 cells. **(A)** Activation and inhibition of ELK1, a down-stream transcription factor of EGFR signaling, was examined using luciferase gene reporter construct (pGL5-luc, 150 ng/well) and with pFA2-Elk1 plasmid (100 ng/well) in A43. The cells were treated with EGF (50 ng/ml) in combination with LEF and TER (1, 5, and 10 μM), and the reference compounds erlotinib (an EGFR inhibitor) and U0126 (an up-stream ERK1/2 kinases inhibitor, both at 10 μM concentration), phenobarbital (500 μM), dexamethasone (100 nM) and DMSO (1‰) in the control samples for 24 h. Luminescence activity was measured in cell lysate using the Dual Luciferase Reporter Assay. Data are presented as the relative activation of firefly luciferase activity normalized to Renilla and related to the EGF-treated controls, set to 100%. **(B)** The interaction of LEF and TER with EGFR was tested in A431 cells treated with EGF. Total EGFR and phosphorylated Y1068 EGFR were measured using the sandwich ELISA method according to the manufacturer’s protocol. The A431 cells were pre-treated for 30 min with the tested compounds (concentrations 1, 5, and 10 μM), erlotinib (10 μM), phenobarbital (500 μM), CITCO (10 μM) and DMSO (0.1%), and then treated with EGF (50 ng/ml) for an additional 10 min. The signal of the phosphorylated protein was normalized with the total EGFR protein, and the background signal was subtracted. The data are expressed as the relative activation related to the cells treated only with EGF (set to be 100%). Data are presented as the mean ± SD of three independent assays (*n* = 3). ^∗^*P* < 0.05, ^∗∗^*P* < 0.01, ^∗∗∗^*P* < 0.001 – statistically significant difference compared to the control vehicle- and EGF-treated cells; *^ff^P* < 0.01 – statistically significant effect of EGF compare to cells without (w/o) EGF treatment (ANOVA with Dunnett’s *post hoc* test).

In our next experiment, we sought to verify if LEF and TER inhibit EGFR and ELK1 activation using a reporter gene assay and EGFR autophosphorylation ELISA assay in A431 cells activated by EGF treatment. The ELK1 transcription factor is an important down-stream factor activated by the EGFR-RAS-RAF-ERK1/2-MEK signaling cascade (Rojas et al., 1996). In addition, ELK1 was demonstrated to regulate hCAR (*NR1I3*) gene expression through a serum responsive element (Osabe and Negishi, 2011). As our data show, both LEF and TER interfered with the activation of ELK1 in a dose-dependent fashion (**Figure 5A**). Erlotinib, an EGFR receptor antagonist, as well as U0126, an ERK1/2 kinase inhibitor, abolished ELK1 activation by EGF. Similar results were obtained in HepG2 cells (*data not shown*). Unfortunately, a concentration of LEF and TER higher than 10 μM significantly inhibited the proliferation of A431 cells, therefore we used concentrations of up to 10 μM in these experiments. Dexamethasone (100 nM) did not suppress or activate ELK1 transcription factor activation indicating that glucocorticoid activity does not interfere with the signaling (**Figure 5A**).

In the following experiments, we studied EGFR phosphorylation at site Y1068 using an ELISA sandwich assay. The binding of EGF to EGFR triggers the autophosphorylation at the tyrosine residue, leading to the pathway activation. We found that LEF and TER strongly interfered with the phosphorylation of EGFR (**Figure 5B**). Similarly, erlotinib and phenobarbital significantly inhibited EGFR receptor activation by EGF (**Figure 5B**). Interestingly, CITCO also significantly abrogated EGFR phosphorylation by 50% at 10 μM concentration (**Figure 5B**).

### Leflunomide and Teriflunomide Activate Glucocorticoid Receptor

**FIGURE 6:**
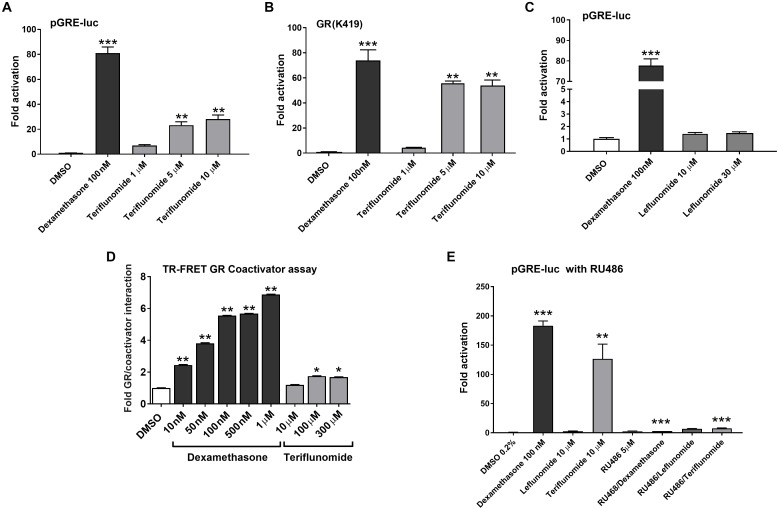
Teriflunomide but not leflunomide activates GR. Wild type GR **(A,C,E)** and stable GR mutant (GR K419) **(B)** were transfected into HepG2 cells together with the reporter plasmid pGRE-luc. LEF and TER were tested at concentrations 1, 5, 10, and 30 μM with the positive control dexamethasone 100 nM and vehicle (DMSO, 0.1%) in the control samples. In experiments in **(E)**, a glucocorticoid antagonist RU486 (5 μM) was used in combination with dexamentasone (100 nM), TER and LEF (10 μM) in HepG2 cells cotransfected with pGRE-luc (150 ng/well) and pSG5-GR (150 ng/well) vectors. Relative activation was calculated to vehicle-treated samples and data are presented as the mean ± SD from three independent experiments (*n* = 3) performed in triplicates. **(D)** LanthaScreen^®^TR-FRET GR Coactivator Binding Assay with recombinant GR LBD was performed according to the manufacturer’s protocol. Data are presented as the relative activation to the control (DMSO-treated sample) from the mean ± SD of three independent assays (*n* = 3) performed in quadruplicates and compared to the vehicle sample (set to 1). ^∗^*P* < 0.05, ^∗∗^*P* < 0.01, ^∗∗∗^*P* < 0.001 – statistically significant difference compared to the control (DMSO-treated) cells (ANOVA with Dunnett’s *post hoc* test).

Next, we aimed to determine if LEF and TER could interact with several other nuclear receptors in cytochrome P450 genes regulation. We found that TER, but not LEF, activates the GR in HepG2 cells transiently transfected with pGRE-luc construct and wild-type human expression construct for GRα (**Figures 6A,C**) or its GR K419 mutant with prolonged degradation (**Figure 6B**). To complement these data with *in silico* noncellular assays, we performed a TR-FRET GR assay with recombinant GR LBD protein. We observed the direct activation of GR LBD by TER in the assay, although the activity of TER to activate GR LBD was much lower than in the cellular assays (**Figure 6D**). The activation of GR-responsive luciferase construct by TER was abolished by RU486 (5 μM), an inhibitor of GR (**Figure 6E**). We also found that LEF significantly activated the aryl hydrocarbon receptor (AHR) in a dose-dependent manner, whereas TER did not show this activity (**Supplementary Figure S2**). Neither LEF nor TER interacted with the vitamin D receptor (VDR), another member of the NR superfamily, which is involved in CYP3A4 and CYP2B6 regulation (*data not shown*).

### Leflunomide and Teriflunomide Affect the Expression of Key Enzymes Controlling Gluconeogenesis and Fatty Acids Synthesis

**FIGURE 7:**
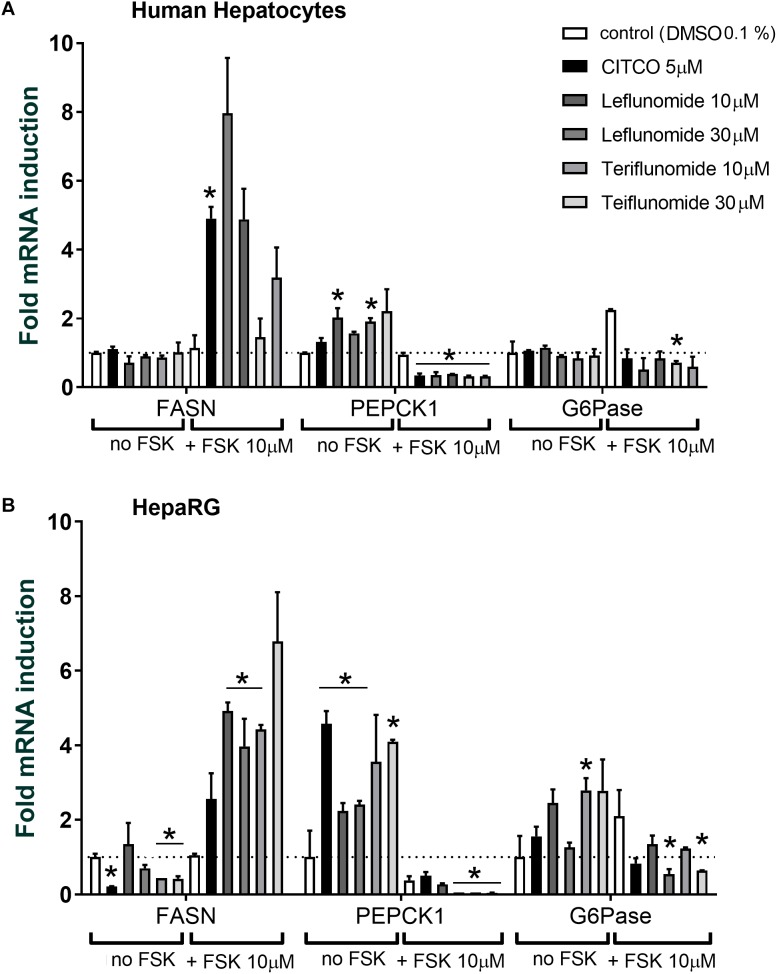
Expression of FASN, PEPCK1, and G6Pase genes mRNAs after treatment with leflunomide and teriflunomide in human hepatocytes **(A)** and HepaRG cells **(B)**. Cells were either pre-treated or not with forskolin (FSK, 10 μM) for 1 h and then treated with CITCO, leflunomide or teriflunomide at concentrations 10 and 30 μM, and vehicle, for 48 h before the isolation of mRNA and qRT-PCR analyses. Data are presented as the mean ± SD of fold mRNA induction from three independent cell cultures (*n* = 3) and compared to the vehicle(DMSO)-treated samples with or without FSK treatment (set to 1). ^∗^*P* < 0.05 – statistically significant difference compared to the control (DMSO-treated) cells with or without FSK pre-treatment (ANOVA with a Dunnett’s *post hoc* test).

Since CAR also regulates critical enzymes controlling gluconeogenesis, fatty acids and steroids synthesis, we studied the effects of LEF and TER on FASN, *PEPCK1*, and G6Pase, *G6PC*, and 3-hydroxy-3-methylglutaryl-CoA synthase 2 (*HMGCS2*) genes in primary human hepatocytes. We found the down-regulation of both G6Pase and PEPCK1 in the primary human hepatocytes (**Figure 7A**) and in the HepaRG cells (**Figure 7B**) pre-treated with FSK after treatment with LEF and TRM in comparison with the vehicle-treated cells pre-treated with FSK. In absence of FSK, both PEPCK1 and G6Pase mRNA expression was up-regulated by LEF and TER treatment in HepaRG cells (**Figure 7B**) and PEPCK1 was up-regulated in the primary human hepatocytes (**Figure 7A**). All the tested compound up-regulated FASN mRNA under FSK-treated conditions, but in the absence of FSK only TER and CITCO displayed a degree of suppressive effect on FASN mRNA expression in the HepaRG cells (**Figure 7B**). We observed no significant effect from either LEF or TER on HMGCS2 expression in the hepatocytes or HepaRG cells (*data not shown*).

We can thus conclude that TER could affect the expression of important genes involved in both detoxification (CYP2B6, CYP3A4, and CYP2C9) as well as gluconeogensis and fatty acid synthesis (PEPCK1 and FASN) in the hepatocyte cellular models where PGC1α signaling is stimulated by FSK.

## Discussion

In this work, we identified the antirheumatic agent LEF and its metabolite TER as inducers of *CYP2B6* mRNA in primary human hepatocytes. Mechanistic inspection revealed that TER is a PB-like indirect activator that promotes the translocation of hCAR into the nucleus and controls its target genes regulating both xenobiotic metabolism as well as endogenous glucose and lipid metabolism. Consistently, in the gene reporter assay, neither LEF nor TER activated hCAR LBD. These data were confirmed by a TR-FRET CAR coactivator assay with recombinant CAR LBD and in a silico docking simulation (**Figures 3C**, **4**). In agreement with the suppressive effect of EGF on hCAR expression (Osabe and Negishi, 2011; de Boussac et al., 2018), TER, an EGFR antagonist, significantly up-regulated CAR expression in differentiated HepaRG cells and in primary human hepatocytes. In addition, we identified TER as a GR ligand, which indicates the third molecular mechanism of TER-mediated hCAR activation through the glucocorticoid-mediated up-regulation of hCAR.

Phenobarbital has been known as an inducer of drug detoxification enzymes for more than 50 years. The discovery of CAR and its interaction with the phenobarbital-responsive element (PBREM) of the *Cyp2b10* gene enhancer provided the first mechanistic explanation suggesting CAR receptor as the PB target transcription factor (Honkakoski et al., 1998). Nevertheless, it soon became evident that phenobarbital is not a ligand of either mouse Car (mCar) or human CAR (hCAR) receptors. However, it was found to stimulate mCar translocation and to induce *Cyp2b10* mRNA in mouse hepatocytes by an alternative indirect mechanism that involves cellular signaling (Kawamoto et al., 1999; Moore et al., 2000; Tzameli et al., 2000). Very recently, breakthrough discoveries by Dr. M. Negishi’s group identified PB as an antagonist of EGF signaling by direct binding to the epidermal growth factor receptor (EGFR). PB thus competitively reverses the EGF signal at the EGF receptor, triggers dissociation of ERK1/2 from CAR homodimer, and converts it into monomer eligible for desphosphorylation at Thr^38^. On the other hand, the direct agonist CITCO binds directly to the hCAR homodimer and dissociates phosphorylated CAR into its monomers, facilitating dephosphorylation at Thr^38^ by PP2Ac. Thus, homodimer-monomer conversion, the initial step in CAR activation, is triggered differently by direct or indirect CAR activators. It has consistently been shown that erlotinib, another prototype EGFR antagonist, dissociates the CAR homodimer, dephosphorylates CAR and significantly induces *Cyp2b10* mRNA in primary mouse hepatocytes (Shizu et al., 2017) and in primary human hepatocytes (**Supplementary Figure S1**), although erlotinib is not a potent direct ligand of CAR LBD (**Supplementary Figure S1**).

Currently, only few known indirect CAR activators have been investigated with respect to CAR translocation or ERK signaling interference. Following PB, phenytoin has been described as an activator of both human and mouse CAR in translocation assays as well as in the induction of *CYP2B6* mRNA in hepatocytes, although direct interaction with human CAR has not been observed (Wang et al., 2004).

Further studies by Wang et al. have identified carbamazepine, efavirenz, nevirapine, chlorpromazine, diazepam, methadone and nefazodone as clinically used drugs capable of inducing hCAR translocation from the cytoplasm to the nucleus, although they do not significantly activate the CAR3 variant or CAR alanine mutant in cell-based reporter gene assay (Faucette et al., 2007; Li et al., 2009; Tolson et al., 2009; Lynch et al., 2013). In addition, the endocrine and metabolism disrupting chemical pollutants chlordane, trans-nonachlor, PCB-126, PCB-153, as well as the atrazine and polychlorinated biphenyls mixture Aroclor 1260 have all been recently proposed as potent EGFR inhibitors and indirect mCAR activators inducing *Cyp2b10* mRNA *in vivo* (Hardesty et al., 2017, 2018). Our group have reported a phenobarbital-like activation of CAR by several flavonoids found in dietary plants in a previous paper (Carazo Fernandez et al., 2015).

Even though several indirect CAR activators have been described, it is not known if they can regulate the same sets of genes as direct CAR agonists. Indirect CAR activators clearly induce classic CAR target genes of the cytochrome P450 enzymes family such as *CYP2B6* and *CYP3A4*. However, little is known regarding whether indirect CAR activators regulate genes involved in the intermediary metabolism of lipids and sugars. Recently, the genome-wide study in HepaRG KO CAR cells found that CITCO and phenobarbital affect the transcriptome of HepaRG differently (Li et al., 2015).

In this work, we studied the effect of LEF and TER on the expression of key enzymes controlling gluconeogenesis (PEPCK1, G6Pase) and fatty acid synthesis (FASN). These gluconeogenic genes are mainly controlled by hepatocyte nuclear factor-4α (HNF4α), cAMP-response element-binding protein (CREB) and forkhead box O1 (FoxO1) transcription factors, PGC1α coactivator, and by GR; PPARα, LXRs, or SREBP-1c are dominant in regulation of lipid metabolism genes (Miao et al., 2006; Oh et al., 2013; Gao et al., 2015). hCAR activation has previously been reported to suppress the expression of gluconeogenic enzymes G6Pase and PEPCK1 in primary human hepatocytes pre-treated with FSK (Gao et al., 2015) and in mice under nutritional stress (high-fat diet, in leptin-deficient obese mice, under fasting conditions) (Miao et al., 2006; Dong et al., 2009; Gao et al., 2009; Yarushkin et al., 2013; Yu et al., 2016). The regulation of the fatty acids and steroids synthetic enzymes HMGCS2 and FASN after treatment with the mCAR agonist TCPOBOP in mice after high- fat or 1% cholesterol diet has been confirmed several times (Gao et al., 2009; Rezen et al., 2009).

In the work, we treated hepatic cells with FSK. FSK directly activates the adenylate cyclase enzyme that increases intracellular cAMP levels and activates CREB, an important regulator of gluconeogenic gene expression. At the same time, PGC1α and FoxO1 expression and activity are augmented after FSK treatment which thus mimics fasting as well as prediabetic state (with elevated PGC1α) in hepatocytes. Under these conditions, PEPCK1 and G6Pase genes are upregulated due to glucagon-stimulated cAMP-dependent signaling or aberant induction of PGC1α (Gao et al., 2015). Nevertheless, FSK is known to affect additional signaling pathways or factors (such as PKA or ERK signaling or PP2A phosphatase) involved in CAR regulation (Sapio et al., 2017). Thus the drawback of the model may be unintentional effects on CAR or on CREB, FoxO1 or HNF4α signaling involved in gluconeogenetic genes regulation.

We found that both TER and LEF affect weakly, but significantly, the expression of the PEPCK1 and G6Pase genes in primary human hepatocytes, suggesting that indirect hCAR activators may have an effect on glucose metabolism (**Figure 7**). Interestingly, we observed PEPCK1 and G6Pase down-regulation after treatment with TER, though we found that TER is a ligand of GR. Glucocorticoids are known to up-regulate PEPCK1 and G6Pase during the hormonal regulation of gluconeogenesis. Thus, we can suppose that a minor overall effect of TER on PEPCK1 and G6Pase expression under FSK-treatment conditions is caused by the opposite glucocorticoid effect of TER.

In fact, the first indirect stimulation of *CYP2B* genes induction via CAR up-regulation was observed in 2000 by Pascussi et al. (2000), who described the GR-mediated regulation of hCAR expression. This finding elucidated hormonal cross-talk between GR and nuclear receptors controlling xenobiotic metabolism, and GR was postulated as an upstream regulator of xenobiotic metabolism. Another noncanonical CAR-related mechanism of CYP3A4 induction via selective induction of CAR expression has been described for the first-generation IGF-1R inhibitor BMS-536924 (Li et al., 2012). We can therefore suppose that TER up-regulates CAR expression in primary human hepatocytes by a novel dual mechanism: by inhibition of the ELK1-mediated suppressive effect on hCAR expression and by transactivation of hCAR through GR activation.

Recently, we also proposed that the classic direct hCAR ligand CITCO could inhibit EGFR autophosphorylation and ELK1 activation at the 10 μM concentration (Carazo Fernandez et al., 2015; **Figure 5B** in the current manuscript). However, a recent paper has suggested no interaction of CITCO with EGFR at the concentration 1 μM (Shizu et al., 2017). Further research should be performed to address these discrepant data and to elucidate the potential dual mechanism of CITCO on CAR activation in higher concentrations.

LEF, an immunosuppressive disease-modifying antirheumatic drug (DMARD), is used in active moderate-to-severe RA and psoriatic arthritis patients with a contraindication or intolerance to MTX. About 70% of the LEF administered converts into TER by a reaction of the opening of the isoxazole ring (**Figure 1A**). TER (A77 1726) is thus the active metabolite of LEF after *per oral* application and it is now registered for the treatment of multiple sclerosis. TER is responsible for the therapeutic actions of LEF mainly via inhibition of the mitochondrial dihydroorotate dehydrogenase (DHO-DH) enzyme. In the human body, TER can interconvert between the *E* and *Z* enolic forms, with the *Z*-enol form being the most stable and predominant (Keen et al., 2013). Interestingly, we observed a very similar effect of LEF in comparison with TER on gene expression (**Figures 1**, **7**), although TER is a more potent inhibitor of EGFR (**Figure 5**) and a ligand for GR (**Figure 6**). This discrepancy can be explained by the rapid conversion of LEF into TER in primary human hepatocytes and in HepaRG cells, which both possess metabolic activities. In contrast, we observed distinct activities between LEF and TER in HepG2 and COS-1 cells, suggesting no interconversion in these metabolic deficient cells **Figures 2**, **6**).

It can thus be concluded that our work has identified and characterized TER as another indirect CAR activator among clinically used drugs. The further study of TER can shed some light onto the physiological and therapeutic functions of CAR in humans. The human CAR is still a great mystery among the NRs superfamily even after more than 25 years since its discovery (Baes et al., 1994). The finding of a specific human CAR ligand or activator would be a milestone that would open a completely new research area regarding CAR.

## Author Contributions

AC, PP, MD, and TSo designed the research. AC, JD, JS, LH, OH, and TSm conducted the experiments. AC and PP wrote the manuscript.

## Conflict of Interest Statement

The authors declare that the research was conducted in the absence of any commercial or financial relationships that could be construed as a potential conflict of interest.

## Abbreviations

CAR: constitutive androstane receptor (NR1I3)
CITCO: 6-(4-chlorophenyl)imidazo[2,1-*b*]thiazole-5-carbaldehyde *O*-(3,4-dichlorobenzyl) oxime
ELISA: enzyme-linked immunosorbent assay
FASN: fatty acid synthase
G6PC: G6Pase, glucose-6-phosphatase
GR: glucocorticoid receptor
LBD: ligand-binding domain
PB: phenobarbital
PEPCK1: phosphoenolpyruvate carboxykinase 1
PP2Ac: protein phosphatase 2Ac
PXR: Pregnane X Receptor
qRT-PCR: quantitative real time – polymerase chain reaction
TR-FRET: time resolved – fluorescence resonance energy transfer.
