# Supplementary material for: Teriflunomide Is an Indirect Human Constitutive Androstane Receptor (CAR) Activator Interacting With Epidermal Growth Factor (EGF) Signaling
Source: Front Pharmacol. 2018 Oct 11;9:993. doi: 10.3389/fphar.2018.00993 (PMC6193428; doi:10.3389/fphar.2018.00993)
Supplement: Supplementary file 1 [file Data_Sheet_1.pdf]

Supplementary data

Carazo et al.

Figure 1S

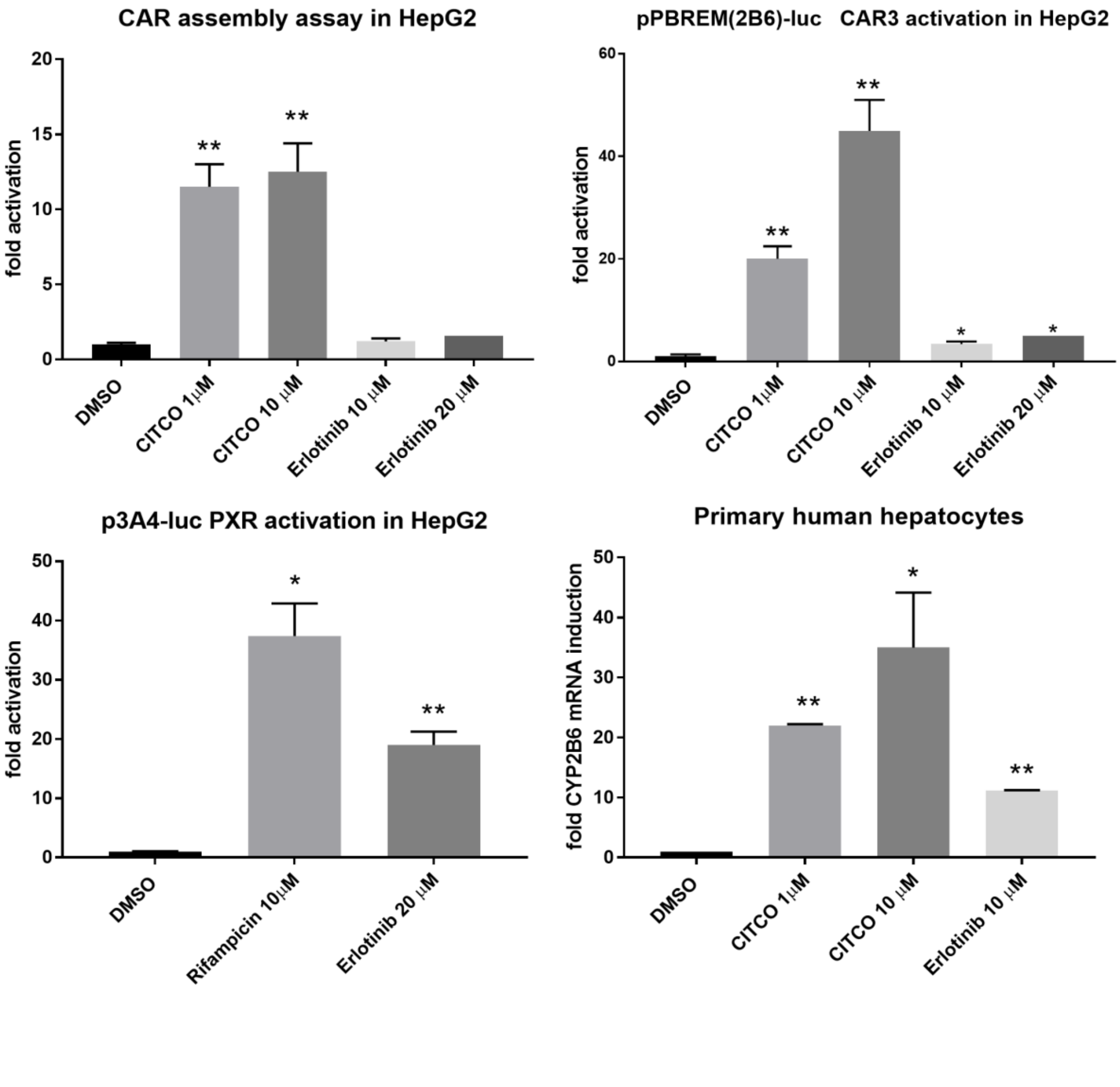

Figure 1S. **Erlotinib is a wear activator of CAR3 variant and PXR ligand.** Luciferase reporter gene assays with CAR- and PXR-responsive promoter constructs have been performed in HepG2 cells treated for 24 h with CITCO, rifampicin and erlotinib. Data are presented as means ±SD and indicate fold activation to vehicle-treated cells. *$P<0.05$, **$P<0.01$, ***$P<0.001$ – statistically significant difference compared to control vehicle-treated cells.

Figure 2S.

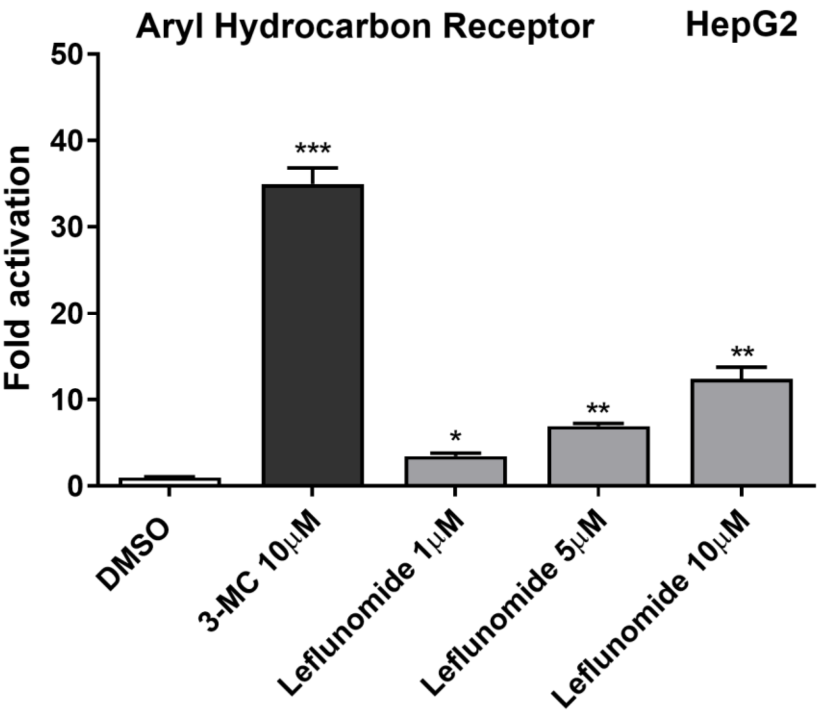

Figure 2S. **Leflunomide activates Aryl hydrocarbon receptor.** HepG2 cells have been transfected with p1A1-luc luciferase reporter AHR-responsive construct. After 24 h, cells were treated with an AHR ligand 3-methylcholantrene (3-MC) or leflunomide (1; 5 or 10 μM) for 24 h. Data are presented as fold activation to vehicle (DMSO)-treated cells. , **P*<0.05, ***P*<0.01, ****P*<0.001 – statistically significant difference compared to control vehicle-treated cells.
